# Effects of SARS-CoV‑2 infections on inpatient mortality of geriatric patients after proximal femoral fracture surgery

**DOI:** 10.1007/s00132-022-04268-z

**Published:** 2022-06-13

**Authors:** Dirk Zajonz, Peter Vaitl, Melanie Edel, Oliver Fuchs, Fabian Kübler, Peter Schneider, Andreas Roth, Torsten Prietzel

**Affiliations:** 1 Department of Orthopedic Surgery, Traumatology and Plastic Surgery, University Hospital Leipzig, Liebigstraße 20, 04103 Leipzig, Germany; 2 ZESBO – Center for Research on Musculoskeletal Systems, Leipzig University, Semmelweisstraße 14, 04103 Leipzig, Germany; 3 Clinic for Orthopedics, Trauma and Reconstructive Surgery, Zeisigwald Hospital BETHANIA, Zeisigwaldstraße 101, 09130 Chemnitz, Germany

**Keywords:** Proximal femoral fracture, Femoral neck fracture, Pertrochanteric femoral fracture, COVID-19, SARS-CoV‑2, Coronavirus disease 2019, Mortality, Geriatrics, Proximale Oberschenkelfraktur, Oberschenkelhalsfraktur, Pertrochanterische Femurfraktur, COVID-19, SARS-CoV‑2, Coronavirus-Krankheit 2019, Sterblichkeit, Geriatrie

## Abstract

**Background:**

The medical challenges caused by severe acute respiratory syndrome coronavirus 2 (SARS-CoV‑2) pose a tremendous burden on the healthcare system. This study aimed to analyze the effects of a SARS-CoV‑2 infections or disease progression on inpatient mortality of geriatric patients after proximal femoral fracture surgery.

**Methods:**

A retrospective analysis was conducted in all patients with a proximal femoral fracture surgically treated in an urban regional trauma center from 01/01/2020 to 01/31/2021. According to PCR test results detecting SARS-CoV‑2, the patients were divided into two groups (SARS-CoV‑2 positive vs. SARS-CoV‑2 negative). Patient data, disease progression data, and treatment-related information were evaluated for all patients. Statistical data analysis was performed using unpaired Student’s *t* test or non-parametric Mann-Whitney *U* test.

**Results:**

A total of 311 patients (women: 70.4%, age: 82.0 ± 11.0 years) were included in this study. Of these 3.9% (12/311) had a positive test result for SARS-CoV‑2. Significantly more deceased patients were found in the group tested positive for SARS-CoV‑2 (SARS-CoV‑2 positive: 41.7%, SARS-CoV‑2 negative: 5.4%, *p* < 0.001). In addition, the number of proximal femoral fracture associated deaths correlated with the number of positive test results performed in the Clinic. The length of stay of SARS-CoV‑2 COVID-19 survivors tended to be greater than in those who were tested negative (SARS-CoV‑2 COVID-19 positive: 15.6 ± 13.1 days, SARS-CoV‑2 COVID-19 negative: 11.5 ± 6.5 days, *p* = 0.683). Furthermore, a significant difference in age was found in SARS-CoV‑2 survivors and SARS-CoV‑2 decedents (deceased: 95.5 ± 7.5 years, alive: 83.5 ± 7.3 years, *p* = 0.020).

**Conclusion:**

The study was conducted before the introduction of SARS-CoV‑2 vaccination. The results therefore refer to immune naive (unvaccinated) patients. In our study, more than 40% of all patients with proximal femoral fractures who tested positive for SARS-CoV‑2 died during hospitalization. An additional, critical factor in this respect was the age of the infected patients. Nonetheless, a positive correlation was demonstrated between the mortality rate and the number of positive SARS-CoV‑2 detections. Regarding the greater length of stay of patients tested positive for SARS-CoV‑2, the limited transfer options (further rehabilitation, skilled nursing facility) of the infected ones can be considered as causal. Particularly the vulnerable older patients are increasingly endangered by a combination of proximal femoral fracture and SARS-CoV‑2.

## Abstract (de)

**Hintergrund:**

Die medizinischen Herausforderungen, die durch das Schwere Akute Respiratorische Syndrom durch Coronavirus 2 (SARS-CoV‑2) verursacht werden, stellen eine enorme Belastung für das deutsche Gesundheitssystem dar. Ziel dieser Studie war es, die Auswirkungen einer SARS-CoV‑2-Infektion oder eines asymptomatischen Krankheitsverlaufs auf die stationäre Mortalität geriatrischer Patienten nach einer proximalen Femurfraktur zu analysieren.

**Methodik:**

Es erfolgte eine retrospektive Analyse aller Patienten mit  proximaler Femurfraktur, die vom 1. Januar 2020 bis zum 31.01.2021 in einem städtischen regionalen Traumazentrum operiert wurden. Anhand der PCR-Testergebnisse zum Nachweis von SARS-CoV‑2 wurden die Patienten in zwei Gruppen eingeteilt (SARS-CoV‑2 positiv vs. SARS-CoV‑2 negativ). Für alle Patienten wurden Patientendaten, Daten zum Krankheitsverlauf und Informationen zur Behandlung ausgewertet. Die statistische Datenanalyse wurde mit dem ungepaarten Student’s t-Test oder dem nichtparametrischen Mann-Whitney-U-Test durchgeführt.

**Ergebnisse:**

Insgesamt wurden 311 Patienten (Frauen: 70,4 %, Alter: 82,0 ± 11,0 Jahre) in die Studie aufgenommen. Von diesen hatten 3,9 % (12/311) ein positives Testergebnis für SARS-CoV‑2. In der positiv auf SARS-CoV‑2 getesteten Gruppe befanden sich signifikant mehr verstorbene Patienten (SARS-CoV‑2 positiv: 41,7 %, SARS-CoV‑2 negativ: 5,4 %, *p* < 0,001). Darüber hinaus korrelierte die Zahl der Todesfälle im Zusammenhang mit proximalen Femurfrakturen mit der Zahl der positiven Testergebnisse in der Klinik . Die Verweildauer der Überlebenden von SARS-CoV‑2 COVID-19 war tendenziell länger als bei denjenigen, die negativ getestet wurden (SARS-CoV‑2 COVID-19 positiv: 15,6 ± 13,1 Tage, SARS-CoV‑2 COVID-19 negativ: 11,5 ± 6,5 Tage, *p* = 0,683). Darüber hinaus wurde ein signifikanter Altersunterschied zwischen den Überlebenden von SARS-CoV‑2 und den Verstorbenen von SARS-CoV‑2 festgestellt (verstorben: 95,5 ± 7,5 Jahre, lebend: 83,5 ± 7,3 Jahre, *p* = 0,020).

**Fazit:**

Die Studie wurde vor der Einführung der COVID-19-SARS-CoV‑2-Impfung durchgeführt. Die Ergebnisse beziehen sich daher auf immun-naive (nicht geimpfte) Patienten. In unserer Studie starben mehr als 40 % aller Patienten mit proximalen Femurfrakturen, die positiv auf SARS-CoV‑2 getestet wurden, während des Krankenhausaufenthalts. Ein zusätzlicher, kritischer Faktor in dieser Hinsicht war das Alter der infizierten Patienten. Dennoch wurde eine positive Korrelation zwischen der Sterblichkeitsrate und der Anzahl der positiven SARS-CoV‑2- nachgewiesen. Hinsichtlich der längeren Verweildauer der positiv auf SARS-CoV‑2 getesteten Patienten können die eingeschränkten Verlegungsmöglichkeiten (Weiterrehabilitation, Fachpflegeeinrichtung) der Infizierten als ursächlich angesehen werden. Insbesondere die vulnerablen älteren Patienten sind durch eine Kombination aus proximaler Femurfraktur und SARS-CoV‑2 zunehmend gefährdet.

## Background

The coronavirus pandemic substantially shaped the year 2020, challenging medicine, politics, and societal structures alike [1]. The global outbreak of the coronavirus disease 2019 (COVID-19), caused by severe acute respiratory syndrome coronavirus 2 (SARS-CoV‑2), represents the third and most devastating pandemic of the twenty-first century to date. Especially in today’s globalized world, the full extent of the damage caused by the rapid spread of a virus pandemic has become apparent [2]. In addition to the drastic impact on social and societal life as well as the global economy, this pandemic has relevant effects on the health sector. In favor of limited resources for SARS-CoV‑2 patients, an elementary part of elective care had to be reduced in almost all specialties, affecting orthopedics and trauma surgery as well [3]. Moreover, new hygiene strategies for testing, isolation, and appropriate treatment of patients attributed to SARS-CoV‑2 had to be both established and integrated into hospital’s tense daily routine [4]. In particular, the burdens of extra work, sickness leave, quarantine, and lack of qualified specialists have exposed the previously underestimated weariness of staff in German hospitals [5, 6]. Ultimately, the economic consequences of this pandemic for many hospitals also cannot be determined clearly because of subsidies paid by the state [7]. In spite of all these unexpected pandemic-related problems, the challenges of emergency medicine, and thus those of trauma surgery, remained mostly unchanged; however, geriatric traumatology takes on an important role, as the elderly are highly vulnerable to SARS-CoV‑2 infections and a severe course of disease is more likely for this group of patients [8]. International studies show a mortality rate of 54% in SARS-CoV‑2 infected inpatients [9]. These observations demonstrate the necessity of examining the disease’s geriatric perspective. Proximal femoral fractures are one of the most common injuries in the elderly [10]. In particular, difficult mobilization of these patients after surgical treatment highly contributes to the occurrence of complications and ultimately to high mortality [11, 12].

This retrospective analysis aimed to analyze inpatient mortality of geriatric patients with surgically treated proximal femoral fractures, focusing on the effects of SARS-CoV‑2 infection or asymptomatic disease progression compared to a control group.

## Methods

All patients provided written informed consent for the analysis and publication of the anonymized data as part of their hospital admission contract. In accordance with the general requirements of good clinical practice, an ethical vote was not obtained for real retrospective studies.

In a monocentric retrospective analysis of an urban primary care facility (local trauma center), all patients who were surgically treated for a proximal femoral fracture between 01/01/2020 and 01/31/2021 were identified. All patients were admitted via the central emergency department and tested for SARS-CoV‑2 using a reverse transcription-polymerase chain reaction (RT-PCR) test. After the establishment of the rapid antigen detection (RAD) test, a rapid test was initially performed (Clongene COVID-19 Antigen Rapid Test, Clungene, Hangzhou Clongene Biotech Co., Ltd.; Paul Ehrlich Institute [PEI], evaluated). In the case of a negative test result, admission was made without extended isolation measures and the test was repeated 3 days later. Otherwise, admission was made to one of our interdisciplinary SARS-CoV‑2 isolation units while the positive RAD test was validated by an additional RT-PCR test on the same day. The tests were performed in a standardized nasogastric manner by trained personnel. Only approved and validated test kits were used. According to the RT-PCR test results, all study participants were divided into the group SARS-CoV‑2 positive or SARS-CoV‑2 negative. For all patients, patient data (e.g., age, sex) and disease progression data such as diagnosis, type of care, length of stay, and mortality rate were recorded. The American Society of Anesthesiologists (ASA) score was used to assess the patient’s preoperative physical status. All data were analyzed using Microsoft Excel version 2013 (Microsoft Corp., Redmond, WA, USA) and SPSS version 27.0 (IBM Corp., Armonk, NY, USA). Statistical analysis was performed using unpaired Student’s *t* test or non-parametric Mann-Whitney *U* test depending on the distribution of data. Results are shown as mean ± SD or median. Pearson’s χ^2^-test was used to assess differences in categorical data. Statistical significant difference was indicated as *p* < 0.05.

## Results

**Fig. 1:**
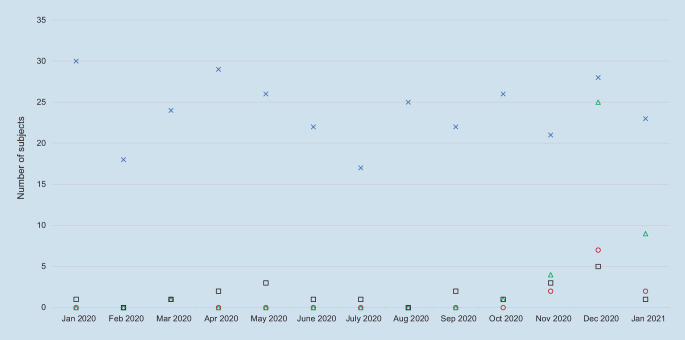
Graphical representation of the development over time of total of proximal femoral fractures (*blue cross*). Number of COVID-19 positive patients with proximal femoral fractures (*red circle*). Number of COVID-19 positive patients (Clinic for Orthopaedics, Trauma and Reconstructive Surgery) (*green triangle*). Total of deceased patients caused by proximal femoral fractures (*black square*)

During the period under consideration, 311 patients with proximal femoral fracture were included in this study. A SARS-CoV‑2 infection was detected in 12 patients (3.9%) on admission and confirmed by RT-PCR testing. All patients were treated surgically according to clinical standards and guidelines. Pertrochanteric or subtrochanteric femoral fractures were usually treated by intramedullary nailing. A cemented or hybrid partial or total hip arthroplasty was performed in patients over 60 years of age with fractured femoral neck, depending on the extent of osteoarthritis and existing complaints prior to the trauma. Study group characteristics can be found in Table 1. On average, approximately 24 (IQR: 17–30) proximal femoral fractures were registered per month. Aside from the usual fluctuations, no significant decline or rise in cases was found concerning the waves of the SARS-CoV‑2 pandemic (Fig. 1). Statistics on inpatient mortality of geriatric patients after proximal femoral fracture surgery during the course of the year (SARS-CoV‑2 positive/negative) can also be found in Fig. 1. Here, a peak was shown in December 2020, which correlates with the number of positive test results for SARS-CoV‑2 (Clinic for Orthopedics, Trauma and Reconstructive Surgery), also peaking in December 2020 (Fig. 1). It turned out that COVID-19 positive patients tended to be older than the other ones (SARS-CoV‑2 positive: 88.5 ± 9.4 years, SARS-CoV‑2 negative: 81.8 ± 11.0 years, *p* = 0.051). It is also striking that the inpatient mortality rate was significantly higher in the SARS-CoV‑2 positive group (41.7%) than in the SARS-CoV‑2 negative group (5.4%). The mean length of stay of survivors suffering from SARS-CoV‑2 was not significantly higher compared to the survivors without SARS-CoV‑2 infections (SARS-CoV‑2 positive: 15.6 ± 13.1 days, SARS-CoV‑2 negative: 11.6 ± 6.7 days, *p* = 0.683). All patients who tested positive for SARS-CoV‑2 and during the hospital stay died, died as a result of pneumonia and subsequent respiratory exhaustion. In contrast, all infection survivors had no or only marginal symptoms. The causes of death of patients without a SARS-CoV‑2 infection are shown in Table 2. Here, two patients also died as a result of pneumonia but repeatedly tested negative for SARS-CoV‑2. A comparative analysis of the deceased and survivors of both groups is shown in Table 3. There was no significant difference in the ASA score assessing the patient’s preanesthesia medical comorbidities or physical constitution; however, it turned out that the deceased patients of the SARS-CoV‑2 positive group tended to receive more general anesthesia (60%) than the infection survivors who received more regional anesthesia (71.4%, *p* = 0.276). The SARS-CoV‑2 negative group showed smaller differences in the anesthesia used during surgery (*p* = 0.593), but without statistical significance. Regarding age, there was a significant difference in the SARS-CoV‑2 positive group. While the survivors had a mean age of 83.5 ± 7.3 years, the deceased were 95.5 ± 7.5 years old (*p* = 0.020) (Table 3).

**Table 1:** Presentation of patient parameters in the study collective and in the individual control groups

	Study group (total)	SARS-CoV‑2 negative	SARS-CoV‑2 positive	*p*-value
Number of subjects	311 (100.0%)	299 (96.1%)	12 (3.9%)	–
Number of male subjects	92 (29.6%)	88 (29.4%)	4 (33.3%)	0.772
Mean age in years (min–max)	82.0 ± 11.0 (46.4–105.7)	81.8 ± 11.0 (46.4–101.6)	88.5 ± 9.4 (69.9–105.7)	0.051
Number of subtrochanteric femoral fractures	17 (5.5%)	17 (5.7%)	0 (0.0%)	0.680
Number of pertrochanteric femoral fractures	156 (50.1%)	150 (50.2%)	6 (50.0%)	
Number of fractures of the femoral neck	138 (44.4%)	132 (44.1%)	6 (50.0%)	
Mean length of stay in days^a^ (min–max)	11.6 ± 6.7 (5–57)	11.5 ± 6.5 (5–57)	15.6 ± 13.1 (7–42)	0.683
Number of deceased patients	21 (6.8%)	16 (5.4%)	5 (41.7%)	*<0.001*
Mean postoperative period to death in days (min–max)	8.4 ± 4.9 (1–20)	8.5 ± 5.6 (1–20)	8.2 ± 1.6 (6–10)	0.851
Median ASA Score^a^ (min–max)	3 (1–4)	3 (1–4)	3 (2–4)	0.990
Median ASA Score of deceased patients (min–max)	3 (2–4)	3 (2–4)	3 (2–3)	0.719

^a^Exclusion of deceased patients

**Table 2:** Cause of death of COVID-19-negative patients absolute and percentage

Cause of death of COVID-19 negative patients	Absolute frequency	Relative frequency (%)
Cardiac decompensation with myocardial failure	7	43.75
Pulmonary embolism	2	12.5
Non-COVID-19 pneumonia	2	12.5
Acute myocardial infarction	1	6.25
Sepsis	1	6.25
Gastrointestinal bleeding	1	6.25
Epileptic shock with aspiration	1	6.25
Hepatic failure	1	6.25

**Table 3:** Comparison of ASA, age and anesthesia procedure between deceased and survivors divided into SARS-Cov 2 positive and negative patients

	SARS-CoV‑2 positive patients (*n* = 12)			SARS-CoV‑2 negative patients (*n* = 299)		
	Deceased	Alive	*p*-value	Deceased	Alive	*p*-value
Number of subjects	5 (41.7%)	7 (58.3%)	–	16 (5.4%)	283 (94.6%)	–
Mean age in years (min–max)	95.5 ± 7.5 (85.1–105.7)	83.5 ± 7.3 (69.9–92.1)	*0.020*	83.5 ± 10.9 (66.2–96.0)	81.7 ± 11.0 (46.4–101.6)	0.324
Median ASA Score (min–max)	3 (2–3)	3 (2–4)	1.000	3 (2–4)	3 (1–4)	0.419
Number of regional anesthesia	2/5 (40.0%)	5/7 (71.4%)	0.276	7/16 (43.8%)	105/283 (37.1%)	0.593

## Discussion

The COVID-19 pandemic is undoubtedly a dramatic global disaster that is also having a fundamental impact on the social and economic life. With over 175 million infected and over 3.8 million deaths attributed to SARS-CoV‑2 (WHO Coronavirus [COVID-19] Dashboard as of 06/13/2021, https://covid19.who.int/), it poses one of the most devastating international medical challenges of the last 100 years after the Spanish flu (1918–1920). Geriatric and multimorbid patients are particularly affected by the serious consequences of the respiratory disease. Thus, the mortality and the risk of severe illness is more likely in the elderly suffering from SARS-CoV‑2 than that of young and middle-aged people [13, 14]. Moreover, these patients suffer from proximal femoral fractures more frequently due to the increased risk of osteoporosis and falls [15]. Since the care of acute trauma patients must also be ensured during shutdown, the combination of a proximal femoral fracture with a SARS-CoV‑2 infection is particularly threatening. Thus, despite the temporary shutdown of the elective surgery in our hospital (03/2020–04/2020, 12/2020, and 01/2021), there was no decline in proximal femoral fractures (Fig. 1). There were 30.0% (12/40) of SARS-CoV‑2 detections within the Department of Orthopedics and Trauma Surgery in patients with proximal femoral fractures peaking during the second wave (11/2020–01/2021) (Fig. 1). Overall, 3.9% (12/311) of patients with proximal femoral fractures suffered from SARS-CoV‑2 infections. All but one were treated during the second wave of the pandemic. It was noticeable that patients who suffered from a SARS-CoV‑2 infections in addition to a proximal femoral fracture showed a significantly higher mortality rate during the inpatient stay compared to the group without an infection (SARS-CoV‑2 positive: 41.7%, SARS-CoV‑2 negative: 5.4%, *p* < 0.001). A study from a clinic in New York was able to prove these numbers in an analysis of their cases from 02/2020 to 04/2020 (35.3% vs. 7.1%) [16]. Moreover, a study group from Greater London confirmed this trend (30.5%, 25/82 vs. 10.3%, 35/340, *p* < 0.001) [17]. A meta-analysis by Wang et al. showed a similar mortality rate of 32% with a relative risk of 5.66 (95% CI: 4.01–7.98, *p* < 0.001) for postoperative mortality in SARS-CoV‑2 positive patients, compared to those who tested negative [18]. It follows that there is a clear consensus that mortality in patients with a proximal femoral fracture is significantly increased by a SARS-CoV‑2 infection. Furthermore, there is a trend regarding prolonged hospitalization of survivors tested positive for SARS-CoV‑2 (SARS-CoV‑2 positive: 15.6 ± 13.1 days, SARS-CoV‑2 negative: 11.5 ± 6.5 days, *p* = 0.683); however, this can be justified by the problematic transfer to skilled nursing facilities or rehabilitation centers (acute geriatrics or geriatric further rehabilitation) of SARS-CoV‑2 positive patients, even if they had no clinical symptoms. Furthermore, the age of the infected patients played a decisive role in the clinical outcomes. The mean age of patients who died in hospital was 95.5 ± 7.5 years, which was significantly higher than the mean age of survivors (83.5 ± 7.3 years) who could be discharged from hospital despite a SARS-CoV‑2 infection (*p* < 0.020). This is supported by the studies from New York and London as well [16, 17].

## Conclusion

The study was conducted before the introduction of COVID-19 vaccination. The results therefore refer to immune naive (unvaccinated) patients. In addition to increased mortality during their inpatient stay, a severe course of disease and longer hospitalization are more likely in elderly SARS-CoV‑2 patients. For this purpose, treatment strategies that meet current needs must be developed, particularly with respect to patient follow-up care. The impact of fully vaccinated or immune competent patients on survival from SARS-Cov‑2 and proximal femoral fractures needs to be clarified in further studies.

### Limitations

False negative tests could have led to an exclusion of asymptomatic COVID-19 cases. Regarding this, it is possible that two patients who died of non-COVID-19 pneumonia were tested false negative for COVID-19 despite the appropriate disease progression (pulmonary manifestation of COVID-19). The missing follow-up after hospitalization also represents a limitation of this study.

## Abbreviations

ASA: American Society of Anesthesiologists
COVID-19: Coronavirus disease 2019
RAD: Rapid antigen detection
RT-PCR: Reverse transcription-polymerase chain reaction
SARS-CoV‑2: Severe acute respiratory syndrome coronavirus 2

## Declarations

For this article no studies with human participants or animals were performed by any of the authors. All studies mentioned were in accordance with the ethical standards indicated in each case. The study was conducted in accordance with the regulations for research on humans according to § 15 paragraph 1 of the Declaration of Helsinki of the World Medical Association in the version of the 64th General Assembly 2013 in Fortaleza. All volunteers provided written informed consent prior to participation in the study, which included anonymous analysis and publication of patient-specific disease data.
